# 4-[Bis(3-phenyl-1*H*-pyrazol-1-yl)meth­yl]benzene-1,2-diol

**DOI:** 10.1107/S1600536811037949

**Published:** 2011-09-30

**Authors:** Florian Blasberg, Hans-Wolfram Lerner, Michael Bolte

**Affiliations:** a Institut für Anorganische Chemie, J. W. Goethe-Universität Frankfurt, Max-von-Laue-Strasse 7, 60438 Frankfurt/Main, Germany

## Abstract

The title compound, C_25_H_20_N_4_O_2_, is a ditopic *ortho*-hydro­quinone-based bis­(pyrazol-1-yl)methane ligand. The dihedral angles between the planes of the pyrazole rings and their attached phenyl rings are 17.4 (3) and 5.9 (4)°. The pyrazole rings make a dihedral angle of 87.84 (16)°. One of the two hy­droxy groups forms an intra­molecular hydrogen bond to the other hy­droxy group, whereas the second is involved in an inter­molecular O—H⋯N hydrogen bond. As a result of these inter­molecular hydrogen bonds, helical chains running along the *b* axis are formed.

## Related literature

For the synthesis, structural characterization and coordination behavior of ditopic *ortho*-hydro­quinone-based bis­(pyrazol-1-yl)methane ligands, see: Blasberg *et al.* (2011 ▶).
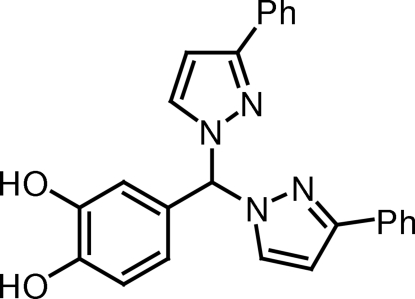


## Experimental

### 

#### Crystal data

C_25_H_20_N_4_O_2_
*M* *_r_* = 408.45
Monoclinic, 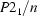

*a* = 13.493 (3) Å
*b* = 5.6288 (11) Å
*c* = 26.309 (5) Å
β = 100.87 (3)°
*V* = 1962.3 (7) Å^3^
*Z* = 4
Mo *K*α radiation
μ = 0.09 mm^−1^
*T* = 173 K
0.40 × 0.15 × 0.10 mm

#### Data collection

Stoe IPDS II two-circle diffractometer
16343 measured reflections
3450 independent reflections
1434 reflections with *I* > 2σ(*I*)
*R*_int_ = 0.111

#### Refinement

*R*[*F* ^2^ > 2σ(*F* ^2^)] = 0.069
*wR*(*F* ^2^) = 0.121
*S* = 0.82
3450 reflections
282 parameters
H-atom parameters constrained
Δρ_max_ = 0.22 e Å^−3^
Δρ_min_ = −0.27 e Å^−3^

### 

Data collection: *X-AREA* (Stoe & Cie, 2001 ▶); cell refinement: *X-AREA*; data reduction: *X-AREA*; program(s) used to solve structure: *SHELXS97* (Sheldrick, 2008 ▶); program(s) used to refine structure: *SHELXL97* (Sheldrick, 2008 ▶); molecular graphics: *XP* (Sheldrick, 2008 ▶); software used to prepare material for publication: *SHELXL97* and *PLATON* (Spek, 2009 ▶).

## Supplementary Material

Crystal structure: contains datablock(s) I, global. DOI: 10.1107/S1600536811037949/ez2261sup1.cif

Structure factors: contains datablock(s) I. DOI: 10.1107/S1600536811037949/ez2261Isup2.hkl

Supplementary material file. DOI: 10.1107/S1600536811037949/ez2261Isup3.cml

Additional supplementary materials:  crystallographic information; 3D view; checkCIF report

## Figures and Tables

**Table 1:** Hydrogen-bond geometry (Å, °)

*D*—H⋯*A*	*D*—H	H⋯*A*	*D*⋯*A*	*D*—H⋯*A*
O23—H23⋯O24	0.84	2.21	2.646 (5)	113
O24—H24⋯N12^i^	0.84	2.08	2.853 (5)	153

Symmetry code: (i) 
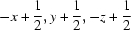
.

**Fig. 1.**
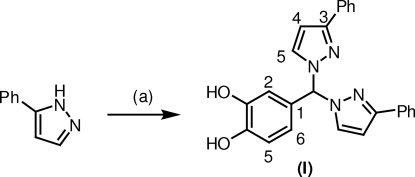
Synthesis and numbering scheme of ortho-hydroquinone-based bis(pyrazol-1-yl)methane ligand (I). (a) (i) NaH, THF, 30 min; (ii) SOCl2, THF, 5 min; (iii) 3,4-dihydroxybenzaldehyde, pyridine, THF, reflux, over night.

**Fig. 2.**
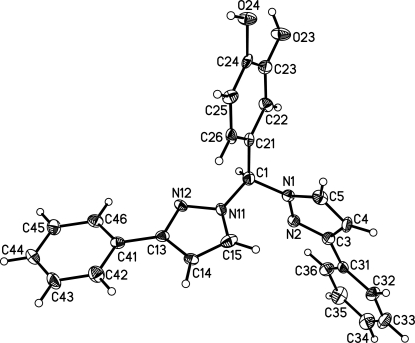
Perspective view of the title compound with displacement ellipsoids drawn at the 50% probability level.

## supplementary crystallographic information

### Comment

Very recently we have reported on the synthesis, structural characterization, and coordination behavior of ditopic *ortho*-hydroquinone-based bis(pyrazol-1-yl)methane ligands (Blasberg *et al.*, 2011). In this report, we have already noted *ortho*-(OH)_2_C_6_H_3_-4-CH(3-Phpz)_2_, but metric parameters will be discussed here. The bis(pyrazol-1-yl)methane derivative (I) was prepared in a three-step one-pot procedure as shown in Fig. 1.

The dihedral angles between the planes of the pyrazol rings and the attached phenyl rings are 20.9 (3)° and 5.9 (4)°. One of the two hydroxy groups forms an intramolecular hydrogen bond to the other hydroxy group, whereas the second one is involved in an intermolecular O—H···N hydrogen bond. As a result of these intermolecular hydrogen bonds, helical chains running along the *b* axis are formed.

### Experimental

Neat 3-phenylpyrazole (2.00 g, 13.87 mmol) was added to NaH (0.33 g, 13.87 mmol) suspended in THF (60 ml) at r.t. After 30 min SOCl_2_ (0.50 ml, 0.83 g, 6.94 mmol) was added in one portion and the resulting mixture stirred at r.t. for 5 min. After 3,4-dihydroxybenzaldehyde (0.96 g, 6.94 mmol) and pyridine (5.60 ml, 4.78 g, 60.40 mmol) were added, the reaction mixture was kept at reflux temperature for 16 h. H_2_O (50 ml) was added and the aqueous phase extracted into CH_2_Cl_2_ (3×50 ml). The combined organic extracts were washed with brine, dried (MgSO_4_), filtered, and the filtrate was evaporated to dryness in vacuo. The crude product was purified by column chromatography (silica gel; CHCl_3_/EtOAc 1:1) and all product-containing fractions were concentrated by rotary evaporation at 40°C to ca. half of the original volume. Upon cooling to r.t. colorless crystals of the title compound precipitated, which were isolated by filtration and washed with Et_2_O. Yield: 1.53 g (54%). Single crystals suitable for X-ray diffraction were obtained by repeatedly dissolving the compound in refluxing MeCN and letting the clear colorless solution cool to room temperature. After three cycles needles of sufficient size were obtained. R</f = 0.63 (silica gel, CHCl_3_/EtOAc 1:1). ^1^H NMR (400.1 MHz, d_6_-DMSO) δ = 6.48 (dd, ^3^J_HH_ = 8.3, ^4^J_HH_ = 2.0, 1 H; HQ—H6), 6.66 (d, ^4^J_HH_ = 2.0, 1 H; HQ—H2), 6.76 (d, ^3^J_HH_ = 8.3, 1 H; HQ—H5), 6.84 (d, ^3^J_HH_ = 2.5, 2 H; pz-H4), 7.31 (m, 2 H; Ph—H4), 7.41 (m, 4 H; Ph—H3), 7.82 (m, 4 H; Ph—H2), 7.91 (s, 1H, CH), 7.94 (d, ^3^J_HH_ = 2.5, 2 H; pz-H5), 9.15 (bs, 2 H; OH). ^13^C NMR (100.6 MHz, d_6_-DMSO) δ = 76.7 (Cpz_2_), 103.5 (pz-C4), 114.4 (HQ—C2), 115.5 (HQ—C5), 118.2 (HQ—C6), 125.3 (Ph—C2), 127.2 (HQ—C1), 127.8 (Ph—C4), 128.7 (Ph—C3), 131.9 (pz-C5), 132.8 (Ph—C1), 145.3, 146.1 (HQ—C3,4), 151.0 (pz-C3). ESI-MS: m/z (%) 263 (67) [M—Phpz]^-^, 408 (100) [M—H]^-^. Anal. Calcd (%) for C_25_H_20_N_4_O_2_ (408.45): C 73.51, H 4.94, N 13.72. Found: C 73.22, H 4.86, N 13.67.

### Refinement

All H atoms were geometrically positioned and refined using a riding model with fixed individual displacement parameters [U(H) = 1.2 *U*_eq_(C) or U(H) = 1.5 *U*_eq_(O, C_methyl_)] using a riding model with O—H = 0.84 Å, C—H(aromatic) = 0.95Å or C—H(methine) = 1.00 Å, respectively. The H—O—C—C torsions angles of the hydroxy groups were refined.

### Crystal data

**Table:**

C_25_H_20_N_4_O_2_	*F*(000) = 856
*M**_r_* = 408.45	*D*_x_ = 1.383 Mg m^−^^3^
Monoclinic, *P*2_1_/*n*	Mo *K*α radiation, λ = 0.71073 Å
Hall symbol: -P 2yn	Cell parameters from 2882 reflections
*a* = 13.493 (3) Å	θ = 3.7–25.8°
*b* = 5.6288 (11) Å	µ = 0.09 mm^−^^1^
*c* = 26.309 (5) Å	*T* = 173 K
β = 100.87 (3)°	Needle, colourless
*V* = 1962.3 (7) Å^3^	0.40 × 0.15 × 0.10 mm
*Z* = 4	

### Data collection

**Table:**

Stoe IPDS II two-circle diffractometer	1434 reflections with *I* > 2σ(*I*)
Radiation source: fine-focus sealed tube	*R*_int_ = 0.111
graphite	θ_max_ = 25.0°, θ_min_ = 3.7°
ω scans	*h* = −16→16
16343 measured reflections	*k* = −6→6
3450 independent reflections	*l* = −31→31

### Refinement

**Table:**

Refinement on *F*^2^	Primary atom site location: structure-invariant direct methods
Least-squares matrix: full	Secondary atom site location: difference Fourier map
*R*[*F*^2^ > 2σ(*F*^2^)] = 0.069	Hydrogen site location: inferred from neighbouring sites
*wR*(*F*^2^) = 0.121	H-atom parameters constrained
*S* = 0.82	*w* = 1/[σ^2^(*F*_o_^2^) + (0.003*P*)^2^] where *P* = (*F*_o_^2^ + 2*F*_c_^2^)/3
3450 reflections	(Δ/σ)_max_ < 0.001
282 parameters	Δρ_max_ = 0.22 e Å^−^^3^
0 restraints	Δρ_min_ = −0.27 e Å^−^^3^

### Special details

**Table:**

Experimental. ;
Geometry. All e.s.d.'s (except the e.s.d. in the dihedral angle between two l.s. planes) are estimated using the full covariance matrix. The cell e.s.d.'s are taken into account individually in the estimation of e.s.d.'s in distances, angles and torsion angles; correlations between e.s.d.'s in cell parameters are only used when they are defined by crystal symmetry. An approximate (isotropic) treatment of cell e.s.d.'s is used for estimating e.s.d.'s involving l.s. planes.
Refinement. Refinement of *F*^2^ against ALL reflections. The weighted *R*-factor *wR* and goodness of fit *S* are based on *F*^2^, conventional *R*-factors *R* are based on *F*, with *F* set to zero for negative *F*^2^. The threshold expression of *F*^2^ > σ(*F*^2^) is used only for calculating *R*-factors(gt) *etc*. and is not relevant to the choice of reflections for refinement. *R*-factors based on *F*^2^ are statistically about twice as large as those based on *F*, and *R*- factors based on ALL data will be even larger.

### Fractional atomic coordinates and isotropic or equivalent isotropic displacement parameters (Å^2^)

**Table:**

	*x*	*y*	*z*	*U*_iso_*/*U*_eq_	
N1	0.6081 (3)	0.2494 (7)	0.30782 (15)	0.0162 (10)	
C1	0.5052 (4)	0.2507 (9)	0.31608 (19)	0.0199 (12)	
H1	0.4873	0.0847	0.3245	0.024*	
N2	0.6784 (3)	0.1200 (7)	0.34051 (15)	0.0181 (9)	
C3	0.7672 (4)	0.1933 (8)	0.33129 (19)	0.0189 (12)	
C4	0.7529 (4)	0.3725 (8)	0.29212 (19)	0.0216 (12)	
H4	0.8037	0.4530	0.2782	0.026*	
C5	0.6523 (4)	0.4033 (9)	0.27902 (19)	0.0224 (12)	
H5	0.6184	0.5127	0.2542	0.027*	
N11	0.4994 (3)	0.4008 (7)	0.36107 (14)	0.0171 (9)	
N12	0.4236 (3)	0.3584 (6)	0.38778 (15)	0.0165 (10)	
C13	0.4318 (4)	0.5389 (8)	0.42204 (18)	0.0171 (12)	
C14	0.5103 (4)	0.6909 (8)	0.4161 (2)	0.0214 (12)	
H14	0.5308	0.8302	0.4356	0.026*	
C15	0.5517 (4)	0.6011 (9)	0.37699 (19)	0.0239 (12)	
H15	0.6064	0.6659	0.3635	0.029*	
C21	0.4313 (4)	0.3296 (8)	0.26841 (19)	0.0179 (12)	
C22	0.4107 (4)	0.1733 (8)	0.22619 (19)	0.0209 (12)	
H22	0.4464	0.0273	0.2272	0.025*	
C23	0.3402 (4)	0.2291 (9)	0.1838 (2)	0.0214 (12)	
O23	0.3195 (3)	0.0753 (6)	0.14321 (13)	0.0287 (9)	
H23	0.2640	0.1116	0.1245	0.043*	
C24	0.2880 (4)	0.4488 (8)	0.18097 (19)	0.0168 (11)	
O24	0.2181 (3)	0.4800 (6)	0.13669 (13)	0.0237 (9)	
H24	0.1816	0.5976	0.1402	0.036*	
C25	0.3102 (4)	0.6034 (8)	0.22209 (18)	0.0196 (12)	
H25	0.2768	0.7525	0.2206	0.024*	
C26	0.3800 (4)	0.5442 (8)	0.26527 (19)	0.0193 (12)	
H26	0.3935	0.6523	0.2935	0.023*	
C31	0.8626 (4)	0.0957 (8)	0.36075 (19)	0.0190 (11)	
C32	0.9534 (4)	0.2161 (8)	0.3623 (2)	0.0238 (13)	
H32	0.9540	0.3577	0.3427	0.029*	
C33	1.0414 (4)	0.1348 (9)	0.3913 (2)	0.0312 (14)	
H33	1.1021	0.2212	0.3919	0.037*	
C34	1.0433 (4)	−0.0750 (10)	0.4203 (2)	0.0317 (14)	
H34	1.1042	−0.1318	0.4409	0.038*	
C35	0.9530 (5)	−0.1962 (9)	0.4177 (2)	0.0332 (15)	
H35	0.9531	−0.3406	0.4364	0.040*	
C36	0.8634 (4)	−0.1155 (9)	0.38900 (19)	0.0249 (13)	
H36	0.8027	−0.2021	0.3884	0.030*	
C41	0.3634 (4)	0.5541 (8)	0.45945 (19)	0.0176 (12)	
C42	0.3673 (4)	0.7591 (9)	0.4918 (2)	0.0263 (13)	
H42	0.4139	0.8830	0.4891	0.032*	
C43	0.3040 (4)	0.7773 (9)	0.5268 (2)	0.0272 (14)	
H43	0.3078	0.9133	0.5485	0.033*	
C44	0.2345 (4)	0.6001 (9)	0.53102 (19)	0.0239 (12)	
H44	0.1905	0.6154	0.5551	0.029*	
C45	0.2296 (4)	0.4007 (9)	0.49981 (19)	0.0248 (12)	
H45	0.1820	0.2790	0.5023	0.030*	
C46	0.2950 (4)	0.3786 (8)	0.46467 (19)	0.0223 (12)	
H46	0.2922	0.2396	0.4440	0.027*	

### Atomic displacement parameters (Å^2^)

**Table:**

	*U*^11^	*U*^22^	*U*^33^	*U*^12^	*U*^13^	*U*^23^
N1	0.019 (3)	0.014 (2)	0.016 (2)	−0.0032 (19)	0.0029 (19)	0.0009 (17)
C1	0.019 (3)	0.021 (3)	0.021 (3)	−0.001 (2)	0.006 (2)	−0.003 (2)
N2	0.019 (2)	0.017 (2)	0.019 (2)	−0.0002 (19)	0.0042 (19)	−0.0018 (18)
C3	0.019 (3)	0.016 (2)	0.022 (3)	0.000 (2)	0.005 (2)	−0.004 (2)
C4	0.016 (3)	0.026 (3)	0.025 (3)	−0.002 (2)	0.010 (2)	0.001 (2)
C5	0.031 (3)	0.018 (3)	0.019 (3)	−0.003 (2)	0.004 (2)	0.004 (2)
N11	0.019 (2)	0.019 (2)	0.016 (2)	−0.005 (2)	0.0077 (19)	−0.0038 (18)
N12	0.021 (3)	0.014 (2)	0.014 (2)	0.0026 (18)	0.0023 (19)	−0.0035 (17)
C13	0.018 (3)	0.019 (3)	0.013 (3)	−0.002 (2)	0.000 (2)	0.002 (2)
C14	0.022 (3)	0.019 (3)	0.023 (3)	−0.005 (2)	0.004 (2)	−0.012 (2)
C15	0.020 (3)	0.022 (3)	0.027 (3)	−0.006 (2)	−0.001 (2)	0.002 (2)
C21	0.015 (3)	0.023 (3)	0.018 (3)	−0.002 (2)	0.008 (2)	0.000 (2)
C22	0.027 (3)	0.015 (2)	0.021 (3)	−0.002 (2)	0.006 (3)	−0.006 (2)
C23	0.021 (3)	0.020 (3)	0.024 (3)	−0.005 (2)	0.005 (2)	−0.009 (2)
O23	0.032 (2)	0.024 (2)	0.025 (2)	0.0058 (18)	−0.0061 (18)	−0.0090 (17)
C24	0.009 (3)	0.020 (3)	0.023 (3)	0.005 (2)	0.005 (2)	0.002 (2)
O24	0.026 (2)	0.0201 (19)	0.022 (2)	0.0082 (16)	−0.0056 (17)	−0.0014 (14)
C25	0.018 (3)	0.016 (2)	0.024 (3)	0.002 (2)	0.000 (2)	0.001 (2)
C26	0.020 (3)	0.017 (3)	0.024 (3)	−0.002 (2)	0.011 (2)	−0.008 (2)
C31	0.020 (3)	0.020 (2)	0.018 (3)	−0.005 (2)	0.008 (2)	−0.007 (2)
C32	0.025 (3)	0.021 (3)	0.025 (3)	−0.010 (2)	0.004 (3)	0.005 (2)
C33	0.020 (3)	0.036 (3)	0.038 (4)	−0.007 (3)	0.008 (3)	0.000 (3)
C34	0.022 (3)	0.037 (3)	0.034 (3)	0.012 (3)	0.002 (3)	0.006 (3)
C35	0.034 (4)	0.027 (3)	0.039 (4)	0.003 (3)	0.007 (3)	0.012 (3)
C36	0.024 (3)	0.020 (3)	0.029 (3)	−0.002 (2)	0.003 (3)	−0.001 (2)
C41	0.020 (3)	0.014 (3)	0.019 (3)	−0.001 (2)	0.001 (2)	−0.002 (2)
C42	0.032 (3)	0.022 (3)	0.027 (3)	−0.001 (3)	0.008 (3)	−0.002 (2)
C43	0.032 (4)	0.025 (3)	0.027 (3)	0.001 (3)	0.012 (3)	−0.010 (2)
C44	0.030 (3)	0.026 (3)	0.017 (3)	0.002 (3)	0.008 (2)	−0.002 (2)
C45	0.026 (3)	0.028 (3)	0.022 (3)	−0.006 (3)	0.010 (2)	0.003 (3)
C46	0.027 (3)	0.016 (2)	0.024 (3)	−0.005 (2)	0.003 (2)	−0.006 (2)

### Geometric parameters (Å, °)

**Table:**

N1—C5	1.360 (6)	C24—C25	1.377 (7)
N1—N2	1.364 (5)	O24—H24	0.8400
N1—C1	1.445 (7)	C25—C26	1.373 (7)
C1—N11	1.468 (6)	C25—H25	0.9500
C1—C21	1.515 (6)	C26—H26	0.9500
C1—H1	1.0000	C31—C32	1.394 (7)
N2—C3	1.333 (7)	C31—C36	1.401 (7)
C3—C4	1.429 (7)	C32—C33	1.364 (7)
C3—C31	1.477 (7)	C32—H32	0.9500
C4—C5	1.348 (7)	C33—C34	1.403 (7)
C4—H4	0.9500	C33—H33	0.9500
C5—H5	0.9500	C34—C35	1.387 (8)
N11—C15	1.354 (6)	C34—H34	0.9500
N11—N12	1.367 (6)	C35—C36	1.376 (7)
N12—C13	1.349 (6)	C35—H35	0.9500
C13—C14	1.392 (7)	C36—H36	0.9500
C13—C41	1.473 (7)	C41—C46	1.376 (7)
C14—C15	1.358 (7)	C41—C42	1.430 (7)
C14—H14	0.9500	C42—C43	1.373 (7)
C15—H15	0.9500	C42—H42	0.9500
C21—C26	1.386 (6)	C43—C44	1.388 (7)
C21—C22	1.403 (6)	C43—H43	0.9500
C22—C23	1.360 (7)	C44—C45	1.385 (7)
C22—H22	0.9500	C44—H44	0.9500
C23—O23	1.362 (6)	C45—C46	1.398 (8)
C23—C24	1.418 (6)	C45—H45	0.9500
O23—H23	0.8400	C46—H46	0.9500
C24—O24	1.365 (6)		
			
C5—N1—N2	111.4 (4)	C25—C24—C23	118.6 (4)
C5—N1—C1	128.0 (4)	C24—O24—H24	109.5
N2—N1—C1	118.7 (4)	C26—C25—C24	120.7 (5)
N1—C1—N11	108.8 (4)	C26—C25—H25	119.7
N1—C1—C21	112.2 (4)	C24—C25—H25	119.7
N11—C1—C21	111.8 (4)	C25—C26—C21	121.2 (4)
N1—C1—H1	107.9	C25—C26—H26	119.4
N11—C1—H1	107.9	C21—C26—H26	119.4
C21—C1—H1	107.9	C32—C31—C36	118.6 (5)
C3—N2—N1	105.2 (4)	C32—C31—C3	120.5 (4)
N2—C3—C4	110.2 (5)	C36—C31—C3	120.8 (5)
N2—C3—C31	120.9 (4)	C33—C32—C31	121.4 (5)
C4—C3—C31	128.8 (5)	C33—C32—H32	119.3
C5—C4—C3	105.5 (5)	C31—C32—H32	119.3
C5—C4—H4	127.2	C32—C33—C34	120.7 (5)
C3—C4—H4	127.2	C32—C33—H33	119.6
C4—C5—N1	107.6 (4)	C34—C33—H33	119.6
C4—C5—H5	126.2	C35—C34—C33	117.5 (5)
N1—C5—H5	126.2	C35—C34—H34	121.3
C15—N11—N12	112.5 (4)	C33—C34—H34	121.3
C15—N11—C1	128.7 (4)	C36—C35—C34	122.6 (5)
N12—N11—C1	118.2 (4)	C36—C35—H35	118.7
C13—N12—N11	103.7 (4)	C34—C35—H35	118.7
N12—C13—C14	110.9 (5)	C35—C36—C31	119.2 (5)
N12—C13—C41	120.5 (4)	C35—C36—H36	120.4
C14—C13—C41	128.7 (4)	C31—C36—H36	120.4
C15—C14—C13	106.7 (4)	C46—C41—C42	118.1 (5)
C15—C14—H14	126.7	C46—C41—C13	122.8 (4)
C13—C14—H14	126.7	C42—C41—C13	119.1 (5)
N11—C15—C14	106.2 (5)	C43—C42—C41	120.1 (5)
N11—C15—H15	126.9	C43—C42—H42	119.9
C14—C15—H15	126.9	C41—C42—H42	119.9
C26—C21—C22	118.5 (5)	C42—C43—C44	121.0 (5)
C26—C21—C1	123.3 (4)	C42—C43—H43	119.5
C22—C21—C1	118.2 (4)	C44—C43—H43	119.5
C23—C22—C21	120.5 (5)	C45—C44—C43	119.5 (5)
C23—C22—H22	119.7	C45—C44—H44	120.3
C21—C22—H22	119.7	C43—C44—H44	120.3
C22—C23—O23	120.3 (5)	C44—C45—C46	119.9 (5)
C22—C23—C24	120.6 (4)	C44—C45—H45	120.0
O23—C23—C24	119.1 (4)	C46—C45—H45	120.0
C23—O23—H23	109.5	C41—C46—C45	121.4 (5)
O24—C24—C25	127.0 (4)	C41—C46—H46	119.3
O24—C24—C23	114.4 (4)	C45—C46—H46	119.3
			
C5—N1—C1—N11	−88.4 (5)	C22—C23—C24—O24	−178.9 (5)
N2—N1—C1—N11	74.6 (5)	O23—C23—C24—O24	1.8 (7)
C5—N1—C1—C21	35.9 (6)	C22—C23—C24—C25	−0.2 (8)
N2—N1—C1—C21	−161.0 (4)	O23—C23—C24—C25	−179.6 (5)
C5—N1—N2—C3	−0.7 (5)	O24—C24—C25—C26	177.4 (5)
C1—N1—N2—C3	−166.4 (4)	C23—C24—C25—C26	−1.1 (8)
N1—N2—C3—C4	0.1 (5)	C24—C25—C26—C21	1.0 (8)
N1—N2—C3—C31	178.5 (4)	C22—C21—C26—C25	0.4 (8)
N2—C3—C4—C5	0.6 (6)	C1—C21—C26—C25	−176.9 (5)
C31—C3—C4—C5	−177.7 (5)	N2—C3—C31—C32	−161.3 (5)
C3—C4—C5—N1	−1.0 (6)	C4—C3—C31—C32	16.8 (8)
N2—N1—C5—C4	1.1 (5)	N2—C3—C31—C36	16.7 (7)
C1—N1—C5—C4	165.2 (4)	C4—C3—C31—C36	−165.2 (5)
N1—C1—N11—C15	34.3 (6)	C36—C31—C32—C33	−1.3 (8)
C21—C1—N11—C15	−90.2 (6)	C3—C31—C32—C33	176.8 (5)
N1—C1—N11—N12	−155.2 (4)	C31—C32—C33—C34	0.6 (9)
C21—C1—N11—N12	80.2 (5)	C32—C33—C34—C35	0.7 (9)
C15—N11—N12—C13	−1.2 (5)	C33—C34—C35—C36	−1.4 (9)
C1—N11—N12—C13	−173.1 (4)	C34—C35—C36—C31	0.7 (9)
N11—N12—C13—C14	0.7 (5)	C32—C31—C36—C35	0.6 (8)
N11—N12—C13—C41	−178.6 (4)	C3—C31—C36—C35	−177.4 (5)
N12—C13—C14—C15	0.0 (6)	N12—C13—C41—C46	5.9 (7)
C41—C13—C14—C15	179.3 (5)	C14—C13—C41—C46	−173.3 (5)
N12—N11—C15—C14	1.2 (5)	N12—C13—C41—C42	−173.9 (4)
C1—N11—C15—C14	172.1 (5)	C14—C13—C41—C42	6.8 (8)
C13—C14—C15—N11	−0.7 (5)	C46—C41—C42—C43	−0.1 (8)
N1—C1—C21—C26	−110.4 (6)	C13—C41—C42—C43	179.7 (5)
N11—C1—C21—C26	12.2 (7)	C41—C42—C43—C44	−0.8 (8)
N1—C1—C21—C22	72.2 (6)	C42—C43—C44—C45	0.7 (8)
N11—C1—C21—C22	−165.1 (5)	C43—C44—C45—C46	0.4 (8)
C26—C21—C22—C23	−1.7 (8)	C42—C41—C46—C45	1.2 (7)
C1—C21—C22—C23	175.8 (5)	C13—C41—C46—C45	−178.6 (5)
C21—C22—C23—O23	−179.1 (5)	C44—C45—C46—C41	−1.4 (8)
C21—C22—C23—C24	1.6 (8)		

### Hydrogen-bond geometry (Å, °)

**Table:**

*D*—H···*A*	*D*—H	H···*A*	*D*···*A*	*D*—H···*A*
O23—H23···O24	0.84	2.21	2.646 (5)	113.
O24—H24···N12^i^	0.84	2.08	2.853 (5)	153.

Symmetry codes: (i) −*x*+1/2, *y*+1/2, −*z*+1/2.
